# Impact of DREAMS interventions on experiences of violence among adolescent girls and young women: Findings from population-based cohort studies in Kenya and South Africa

**DOI:** 10.1371/journal.pgph.0001818

**Published:** 2023-05-10

**Authors:** Elvis Omondi Achach Wambiya, Annabelle J. Gourlay, Sarah Mulwa, Faith Magut, Nondumiso Mthiyane, Benedict Orindi, Natsayi Chimbindi, Daniel Kwaro, Maryam Shahmanesh, Sian Floyd, Isolde Birdthistle, Abdhalah Ziraba

**Affiliations:** 1 School of Health and Related Research (ScHARR), University of Sheffield, Sheffield, United Kingdom; 2 Health and Systems for Health, African Population and Health Research Center, Nairobi, Kenya; 3 Faculty of Epidemiology and Population Health, London School of Hygiene & Tropical Medicine, London, United Kingdom; 4 Kenya Medical Research Institute, Centre for Global Health Research, Kisumu, Kenya; 5 Clinical Research Department, Africa Health Research Institute, Durban, South Africa; 6 Institute for Global Health, University College London, London, United Kingdom; 7 Kenya Medical Research Institute-Wellcome Trust Research Programme, Center for Geographic Medicine Research, Kilifi, Kenya; 8 University of KwaZulu-Natal, KwaZulu-Natal, South Africa; University at Buffalo, UNITED STATES

## Abstract

DREAMS aims to reduce HIV incidence among adolescent girls and young women (AGYW) by tackling drivers of HIV risk including gender-based violence. We evaluate the impact of DREAMS on recent experiences of violence perpetuated by men against AGYW. AGYW cohorts were randomly selected from demographic platforms in South Africa (rural KwaZulu-Natal) and Kenya (Nairobi informal settlements and rural Gem sub-county). AGYW aged 13–22 years were enrolled in 2017 (Nairobi, KwaZulu-Natal) or 2018 (Gem), with annual follow-up to 2019. We described proportions of AGYW who self-reported experiences of violence perpetrated by males in the 12 months preceding the interview, overall and by form (physical, sexual, emotional). We investigated associations with DREAMS (invitation to participate during 2017–2018) through multivariable propensity score-adjusted logistic regression and estimated the causal effect of DREAMS on experiences of violence, under counter-factual scenarios in which all versus no AGYW were DREAMS beneficiaries. Among 852, 1018 and 1712 AGYW followed-up in 2019 in Nairobi, Gem and KZN, respectively, proportions reporting any violence in 2019 were higher in Nairobi (29%) than Gem (18%) and KwaZulu-Natal (19%). By sub-type, emotional and physical violence were more frequently reported than sexual violence. We found no evidence of an impact attributable to DREAMS on overall levels of violence, in any setting. Nor was there evidence of impact on sub-types of violence, with one exception: an increase in physical violence in Nairobi if all, versus no, AGYW were DREAMS beneficiaries (16% vs 11%; +5% difference [95% CI: +0.2%, +10.0%]). Experiences of gender-based violence were common among AGYW, especially in urban settings, and DREAMS had no measurable impact on reducing violence within three years of implementation. Violence prevention programming that reaches more men and the broader community, sustained for longer periods, may yield greater gains in violence reduction than AGYW-focused programming. Additionally, more investment in implementation research is needed to bridge trial-based study findings from efficacy to population-level effectiveness.

## Introduction

Gender-based violence (GBV) against women and girls is a persistent public health threat that puts women’s health and wellbeing at risk and violates their human rights. GBV can take different forms including physical, sexual and emotional abuse [1]. The World Health Organization (WHO) estimated that globally, in 2018, 1 in 3 women ever experienced physical or sexual violence, or both, by an intimate partner or non-intimate male partner, with little change in this proportion over the last decade [1–3]. Evidence points to violence starting early, with 1 in 4 ever-partnered adolescent girls and young women (AGYW) aged 15–24 years globally having already experienced physical or sexual violence by a partner [1,3]. The African region is among the WHO regions with the highest lifetime prevalence of violence, with 36% of women aged 15–49 years ever experiencing physical and/or sexual violence from a partner or non-partner [1,3]. Studies from multiple settings in sub-Saharan Africa (SSA), including Kenya and South Africa, also indicate high levels of exposure to GBV among AGYW [4–12].

Violence against women has far-reaching negative physical, mental, sexual and reproductive health effects. These include physical trauma or injuries; depression, post-traumatic stress and anxiety disorders, and suicide; unintended pregnancies, abortions, miscarriages and still births; and increased risk of acquiring sexually transmitted infections (STIs) [13–17]. Furthermore, a recent Global Burden of Disease study ranks intimate partner violence (IPV) (any behaviour within an intimate relationship that causes physical, psychological or sexual harm to those in the relationship) as the second most common factor contributing to disability-adjusted life years globally in women aged 20–24 years [18,19].

Exposure to GBV has a causal association with incidence of HIV infection [20]. Multiple studies have documented pathways between exposure to violence and women’s vulnerability to HIV [9,16,21,22]. Such pathways include direct infection from forced sexual intercourse and associated genital trauma. Proximate determinants include limited control over the circumstances of sexual intercourse including negotiating condom use, reduced access to HIV prevention services, and characteristics of violent male partners such as having multiple and/or concurrent sexual partners and a higher likelihood of HIV-positivity [7,8,10,23–25]. More distal, structural drivers including poverty and economic stresses, gender inequalities, prevailing social norms, and misogyny, create the conditions that enable violence and HIV acquisition to persist.

Increasing attention has focused on addressing the structural drivers of exposure to violence among women. Recent evidence has shown the potential of interventions combining economic strengthening and gender-transformative programming in reducing IPV and HIV risk behaviours among women [26]. Intervention with Microfinance for AIDS and Gender Equity (IMAGE) combined gender training and community mobilization with microfinance and reduced IPV by 55% among women aged between 15 and 49 in rural South Africa [27]. Building from this design, the MAISHA trial conducted in Tanzania subsequently demonstrated a reduction in physical and/or sexual IPV among women aged 33–46 years [28]. However, the evidence is less clear among young women [29,30]. A government-implemented cash plus intervention in Tanzania that combined life-skills training, mentoring and microfinance was effective at reducing female participants’ experiences of sexual violence and male perpetration of physical violence [31]. While some interventions have shown positive results on reducing IPV and GBV outcomes, others aimed at addressing the structural drivers of exposure have not been effective. For instance, the adolescent girls empowerment program (AGEP) in Zambia and the ‘Girl Empower’ program in Liberia had no effect on the acceptability of IPV and protection from sexual violence, respectively [32,33]. A few interventions have aimed to reduce violence among AGYW, but results have been mixed [29,30]. Furthermore, several interventions reporting reductions in violence, such as IMAGE [34], included AGYW but did not publish age-stratified results for those aged under 25 [30].

The DREAMS (Determined, Resilient, Empowered, AIDS-free, Mentored and Safe girls) Partnership established by the United States President’s Emergency Plan for AIDS Relief (PEPFAR) was developed primarily to reduce HIV incidence among AGYW in 10 SSA countries by tackling the multiple, underlying causes of AGYW vulnerability to HIV. This includes addressing GBV, incomplete schooling and socioeconomic vulnerabilities, and empowering AGYW and their communities through a holistic package of evidence-based biomedical, behavioural and social protection interventions [35]. The DREAMS core package integrates violence prevention education into school-based and community-based interventions, including Start, Awareness, Support, Action (SASA!), Stepping Stones and Vhutshilo, all curriculum based interventions that aim to raise awareness, support and action around violence in the community [34,36–38]. DREAMS also provides post-violence care services, social asset building interventions and other interventions that aim to empower AGYW, strengthen the families of AGYW economically, and mobilize communities [35,39]. These interventions are delivered by DREAMS implementing partners (IPs) who are organizations experienced in providing sexual and reproductive health and/or HIV-related services in the recipient countries. A recent publication reported the uptake of DREAMS interventions in the three study sites [40]. The most accessed interventions in all study sites were HIV testing, social protection, and school-based interventions. There was higher uptake of HIV testing and contraception among older AGYW while younger AGYW more frequently accessed social asset building and school-based interventions. There were differences in uptake by site for interventions such as contraception, parenting and social protection programmes, and the provision of PrEP. Among the DREAMS implementation countries are Kenya and South Africa, which are considered HIV endemic countries where the risk among AGYW is high. Recent evidence from DREAMS program in South Africa shows a relationship between IPV and the HIV care and treatment cascade including awareness of HIV status, ART uptake and viral suppression [41].

Despite increasing evidence of efficacy from trials in controlled conditions, the effectiveness of multi-component interventions addressing violence against AGYW is limited in real-world circumstances [26]. This study assessed the impact of DREAMS on experiences of physical, emotional, and sexual violence among AGYW in three settings in Kenya and South Africa over three years of DREAMS implementation.

## Methods

### Study settings

The evaluation was conducted in an urban setting (informal settlements in Nairobi) and a rural setting (Gem, Siaya county) in Kenya, and a rural setting (uMkhanyakude, KwaZulu-Natal) in South Africa. These settings are characterised by historically high HIV prevalence and incidence [42–47], and were identified as priority districts for DREAMS programme implementation [39]. The study settings also feature long-term health and demographic surveillance systems (HDSS) that have been described in detail elsewhere [39]. Briefly, Korogocho and Viwandani informal settlements constitute the Nairobi Urban Health and Demographic Surveillance System [48]. Gem sub-county is nested within an HDSS in Siaya county of western Kenya [49]. The district of uMkhanyakude is included within an HDSS in northern KwaZulu-Natal [50].

### Evaluation study design and sampling

The DREAMS impact evaluation design has been described in detail in the study protocol [39]. In brief, a prospective cohort study design was used to assess impacts of the non-randomised DREAMS intervention packages on representative samples of adolescent girls and young women. Randomly-selected closed cohorts of AGYW were drawn from the wider HDSS sampling frames and enrolled in 2017 in Nairobi and uMkhanyakude, and 2018 in Gem, stratified by age-group at enrolment (13–17 and 18–22 years in Gem and uMkhanyakude; 15–17 and 18–22 in Nairobi). A baseline survey was administered in 2017 in Nairobi and UMkhanyakude with annual follow-up surveys in 2018 and 2019. In Gem, the baseline survey was conducted in 2018 with a follow-up survey in 2019. For all settings, sample size was calculated to ensure statistical power to detect a difference in impact between DREAMS beneficiaries and non-beneficiaries across different outcomes. A detailed report of the sample size calculations have been presented in the published DREAMS protocol paper [39]. In brief, the sample sizes in UMkhanyakude and Gem to measure changes in HIV incidence were calculated using estimates of HIV incidence reported from HDSS data and powered on the number of person years required to show a 30% reduction in HIV incidence in the overall sample and in subgroup analyses. The estimated sample sizes of 1500 AGYW in Nairobi, 2000 in UMkhanyakude, and 1000 in Gem were considered sufficient for the nested cohorts to detect changes in secondary outcomes (including violence exposure) in the study sites with 20% over-sampling to cater for non-response and LTFU.

### Evaluation study data collection

Data were collected face-to-face using an electronic tool administered by trained and experienced field interviewers who were conversant with the study sites. The field interviewers were recruited based on their academic qualifications, working experience in quantitative and qualitative data collection, fluency in English and Swahili languages, and knowledge of the study sites. The tool collected data on socio-demographic/economic characteristics of AGYW and their households, exposure to DREAMS interventions, sexual behaviour and reproductive health outcomes, and exposure to violence. Prior to implementation, the tool was translated into the local language and back-translated to ensure that questions retained their original meaning, then piloted. Data collection in Nairobi took place during March-July 2017, July-December 2018 and May-August 2019; in Gem during January-October 2018, and January-November 2019; and in uMkhanyakude during May 2017-February 2018, April-August 2018, and May-October 2019. Intensive tracing efforts, including multiple attempts to follow-up participants and provision of refreshments, were made to maximise cohort retention.

### DREAMS core package implementation

DREAMS core package interventions, described in detail elsewhere [51], were introduced in the evaluation settings in 2016 by IPs with experience in providing sexual and reproductive health and/or HIV-related services [52]. Roll-out was staggered and by 2017 all interventions were being implemented. DREAMS interventions were intended for vulnerable AGYW at high risk of acquiring HIV, and IPs adopted different approaches to identify and recruit these individuals to participate in DREAMS. In Kenya, the ‘girl roster’ census method was used to inform these decisions and invite AGYW to DREAMS, for example, those who were out of school, or had a child/were pregnant [39,53]. In uMkhanyakude, AGYW were identified as vulnerable and eligible for DREAMS interventions based on geographic mapping exercises, delivering interventions through longstanding community based organisations familiar with orphans and vulnerable children, vulnerable families and those identified as vulnerable through schools [54].

### Measures

#### Outcome

Questionnaire items measuring experiences of violence among AGYW were adapted from the World Health Organisation violence against women instrument [55]. These were 15 questions (yes/no) that asked whether the participant experienced any of the 15 acts of violence by a man in the 12 months preceding the survey. The 15 items were grouped into three forms of violence: emotional violence (items 1 to 3), physical violence (items 4 to 11), and sexual violence (items 12 to 15) [56]. Separate binary outcomes were defined for each form of violence, whereby an experience of violence was defined as yes to any of the applicable questions, for example, yes to any of items 1–3, for psychological violence. An outcome that measured exposure to any form of violence (either physical, psychological or sexual violence, i.e., yes to any of the 15 questions) was also constructed.

#### Participation in DREAMS intervention and confounders

Exposure to DREAMS was defined based on participants’ responses to the question “have you been invited to participate in the DREAMS program?” (yes/no). The primary exposure variable constructed was ‘invitation to DREAMS by 2018’, for which AGYW were categorized as DREAMS beneficiaries if they answered ‘yes’ to the question in 2017 or 2018, and non-beneficiaries if they answered ‘no’ in both 2017 and 2018. For cases where AGYW were followed-up in 2019 but not in 2018, invitation by 2018 was inferred from invitation status reported in 2017 and 2019.

Directed acyclic graphs (DAGs) were constructed to hypothesize the causal relationship between being a DREAMS beneficiary and exposure to violence, informed by the researchers’ understanding of context, DREAMS targeting and implementation–based on discussions with DREAMS implementing partners and review of existing literature. DAGs provide a visual representation of causal effects between variables to reduce bias when conditioning on covariates and are increasingly being used in epidemiological studies [57–59]. In the DAG approach, arrows connecting two variables indicate a causal relationship whereas variables with no direct causal relationship are left unconnected. Through this process, a minimum set of confounders was identified for each study setting, and included such factors as: age at cohort enrolment, rural/ urban residence, ethnicity, marital status, orphanhood, level of education completed, whether currently in school, migration status, ever had sex, ever pregnant, food insecurity, and wealth index (constructed using principal components analysis using information on household and individual assets, household structure, water supply and sanitation).

### Data analysis

Characteristics of AGYW at the time of enrolment were summarized by age group and invitation to DREAMS using counts and proportions. The proportions of AGYW reporting experience of different forms of violence were summarized at each round of follow-up to identify changes over time.

To assess the impact of DREAMS on experience of violence, a staged approach was used. Firstly, multivariable logistic regression models were fitted to assess the associations between exposure to DREAMS (invited by 2018) and violence outcomes measured in 2019, adjusting initially for age (and informal settlement area in Nairobi), then for all potential confounders identified from the DAGs. Unadjusted, age/site-adjusted and fully adjusted odds ratios (OR) and 95% confidence intervals (CI) were reported.

We then applied a causal inference framework using propensity score regression adjustment to control for confounding and to compare counterfactual scenarios. The propensity scores (the probability of being a DREAMS beneficiary given individual and household characteristics) were generated through a logistic regression model with invitation to DREAMS as the outcome and the explanatory variables being characteristics identified from the DAGs. This step ensures that the analysis accounts for different socio-demographic characteristics of AGYW that may influence DREAMS participation. After the propensity score estimation, logistic regression models were fitted separately for each violence outcome for AGYW who were DREAMS beneficiaries and non-beneficiaries, adjusting for the propensity scores and age group. The two logistic regression models were then used to predict the probability of experiencing the different forms of violence under a scenario where none of the AGYW was a DREAMS beneficiary and another scenario where they were all a DREAMS beneficiary. The measure of causal effect was the difference in the average predicted probability between the two hypothetical scenarios: one in which no individuals were a DREAMS beneficiary, the other in which all individuals were a DREAMS beneficiary. Confidence intervals were generated using a bootstrap procedure, repeating the estimation procedure described above in 1000 samples taken with replacement from the complete dataset, and calculating 95% CIs from the resulting bootstrap distribution using the 2.5% and 97.5% percentiles. Sensitivity analyses were also conducted using alternative approaches to control for confounding: inverse-probability-of-treatment (IPTW) weighting (with probability of treatment equal to the propensity score), stratification on the propensity score, and predictions using multivariable logistic regression models of the outcome on confounding variables.

All analyses were restricted to AGYW followed-up in 2019, and were conducted using Stata (StataCorp, College Station, TX) software.

### Ethical considerations

Ethics approval was obtained from AMREF Health Africa, the Kenyan Medical Research Institute, the University of KwaZulu-Natal, and the London School of Hygiene & Tropical Medicine. Interviewers discussed information sheets with potential participants and their parents/guardians and took written informed consent, or for example, thumb prints for those unable to sign. For participants aged below 18 years, assent was obtained from the minor before the parent/ guardian gave consent for participation. All participants were screened for emotional distress immediately following the survey. Those who reported experiences of violence or post-traumatic distress were referred to local DREAMS partner organizations within the study sites that provided post-violence care. The study coordinator followed-up with the organizations regularly to ensure that any cases received appropriate care.

## Results

### Cohort retention

**Fig 1:**
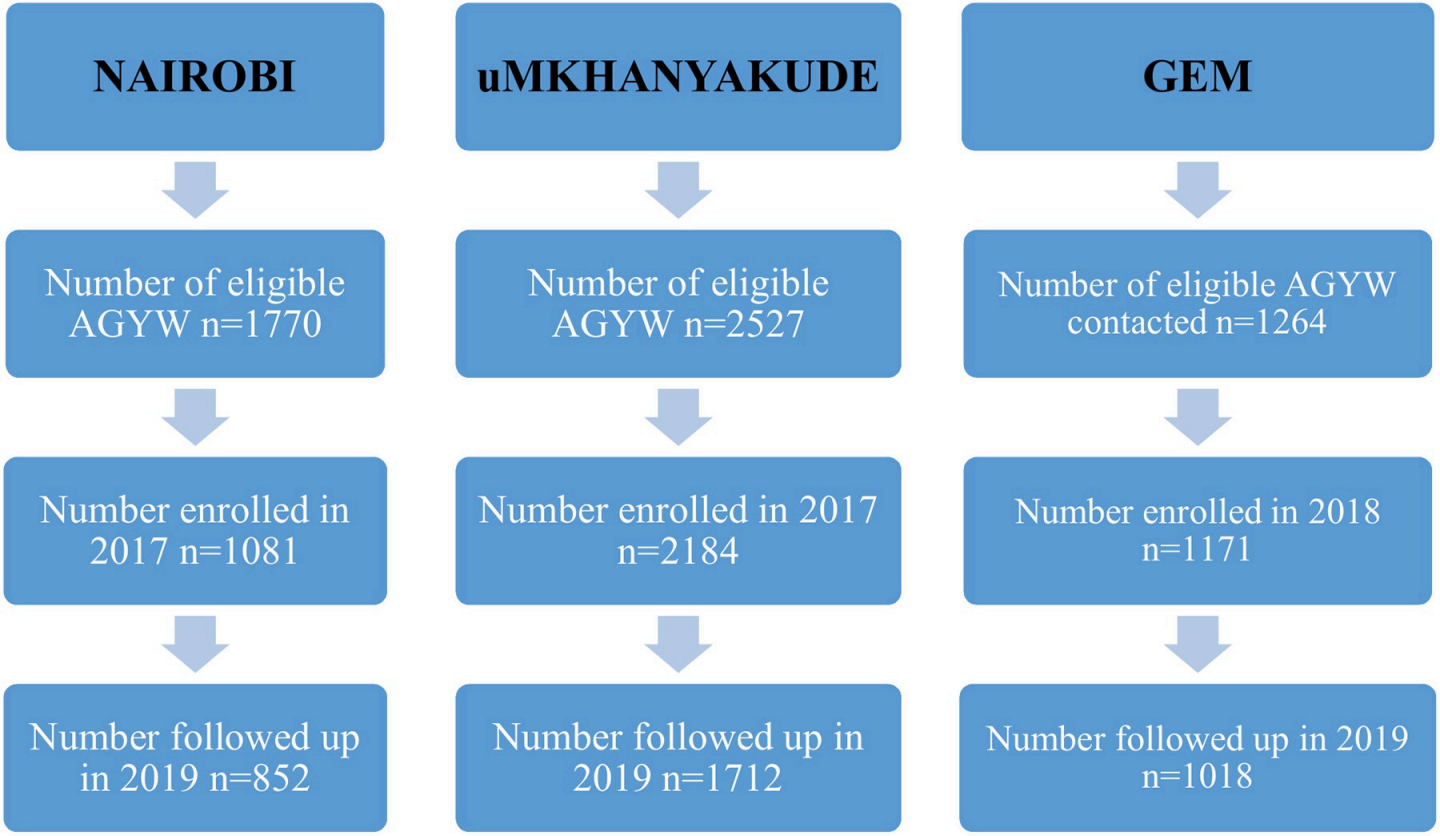
Cohort flow diagrams of AGYW aged 13–22 years followed up between 2017 and 2019 in Nairobi, uMkhanyakude and Gem.

In Nairobi, 852 AGYW aged 15–22 years were followed-up in 2019 out of 1081 enrolled into the cohort in 2017 (79% retention rate). In uMkhanyakude, 1712 AGYW were followed-up in 2019 out of 2184 enrolled in 2017 (78%). In Gem, 1018 AGYW were followed-up out of 1171 enrolled in 2018 (87%). Cohort flow diagrams are presented in **Fig 1.** The main reasons for loss to follow-up in all settings were out-migration from the enumeration area and participants not being reached.

**S1–S3 Tables** show loss to follow up (LTFU) status of AGYW by individual characteristics at enrolment in Nairobi, Gem, and uMkhanyakude. AGYW who were invited to DREAMS in 2017, and in school (Nairobi and uMkhanyakude) or with secondary education (Gem), were more likely to be followed-up in 2019. Those who were older at enrolment and had ever had sex or a pregnancy (Kenyan settings), were less likely to be followed-up. In Nairobi, AGYW who reported any recent violence in 2017 were less likely to be followed-up in 2019 than those who had not experienced violence, while there were no differences in LTFU by violence in Gem or uMkhanyakude.

### Characteristics of AGYW

Overall characteristics at enrolment of AGYW who were followed-up in 2019 are shown in **Table 1**. Higher proportions of younger than older AGYW were enrolled in Nairobi (55% vs 46%), Gem (61% vs 39%) and UMkhanyakude (57% vs 43%). The majority of AGYW in Nairobi (63%) and UMKhanyakude (79%) were in school. About one third of AGYW in all sites reported being food insecure (34% in Nairobi, 23% in Gem, 31% in UMkhanyakude). While socioeconomic status (SES) was evenly distributed in Nairobi, there were more AGYW in the low SES group in Gem (42%) and UMkhanyakude (36%). Characteristics of AGYW stratified by whether they were a DREAMS beneficiary by 2018 are presented in **S4 Table**. The majority were classified as DREAMS beneficiaries (74% Nairobi; 57% Gem; 53% uMkhanyakude). High proportions were in school, more so among DREAMS beneficiaries compared to non-beneficiaries (68% vs 51%, Nairobi; 88% vs 69%, uMkhanyakude). DREAMS beneficiaries were also younger compared to non-beneficiaries (e.g., 59% vs 42% were aged 15–17 in Nairobi), more frequently reported food insecurity (e.g., 26% vs 17% in Gem) were of lower socio-economic status (e.g., 39% vs 32% low SES, uMkhanyakude), and had never had sex (e.g., 69% vs 56% Nairobi).

**Table 1:** Characteristics of DREAMS beneficiaries and non-beneficiaries at time of cohort enrolment in Nairobi, Gem and uMkhanyakude.

Characteristics at enrolment	Nairobi N = 852 n (%)	Gem N = 1018 n (%)	uMkhanyakude N = 1712 n (%)
**Age group**			
13–17		622 (61.1)	972 (56.77)
15–17	464 (54.5)		
18–22	388 (45.5)	396 (38.9)	740 (43.23)
**Age, median (IQR)**	17 (16–19)	16 (14–19)	17 (15–19)
**Site**			
Korogocho	513 (60.2)	-	-
Viwandani	339 (39.8)	-	-
**Geographic area**			
Rural	-	-	1095 (64.49)
Peri-urban/ urban	-	-	603 (35.51)
**Highest level of education**			
None/incomplete primary	92 (10.8)	435 (42.7)	176 (10.29)
Complete primary	170 (20)	-	-
Some secondary	410 (48.1)	-	1323 (77.32)
Complete Sec/Tertiary	180 (21.1)	372 (36.5)	212 (12.39)
Unknown	-	211 (20.7)	-
**Schooling status (currently in school)**			
No	312 (36.6)	-	359 (20.97)
Yes	540 (63.4)	-	1353 (79.03)
**Food insecurity**			
No	563 (66.1)	789 (77.5)	1175 (68.83)
Yes	289 (33.9)	229 (22.5)	532 (31.17)
**Socio-economic status**			
Low	303 (35.56)	424 (41.7)	592 (35.94)
Middle	277 (32.51)	195 (19.2)	576 (34.97)
High	272 (31.92)	399 (39.2)	479 (29.08)
**Marital status**			
never married	695 (81.6)	-	-
Married/living with partner	157 (18.4)	-	-
**Migrated/ moved**			
No	-	-	1432 (83.64)
Yes	-	-	280 (16.36)
**Orphanhood status**			
Not an orphan	663 (77.8)	615 (60.4)	-
Single/double orphan	189 (22.2)	160 (15.6)	-
Unknown	-	243 (23.9)	-
**Pregnancy history**			
No	647 (75.9)	859 (84.4)	1275 (75.22)
yes	205 (24.1)	159 (15.6)	420 (24.78)
**Sexual history**			
No	557 (65.4)	701 (68.9)	1063 (63.35)
Yes	295 (34.6)	317 (31.1)	615 (36.65)
**Experienced any violence**			
No	500 (58.7)	839(82.4)	1126 (65.8)
Yes	352 (41.3)	179(17.6)	586 (34.2)
**Experienced Physical violence**			
No	664 (77.9)	907(89.1)	1243 (72.6)
Yes	188 (22.1)	111(10.9)	469 (27.4)
**Experienced emotional violence**			
No	581 (68.2)	922(90.6)	1373 (80.2)
Yes	271 (31.8)	96(9.4)	339 (19.8)
**Experienced Sexual violence**			
No	728 (85.5)	965(94.8)	1565 (91.4)
Yes	124 (14.5)	53(5.2)	147 (8.6)

### Levels of violence

**Fig 2:**
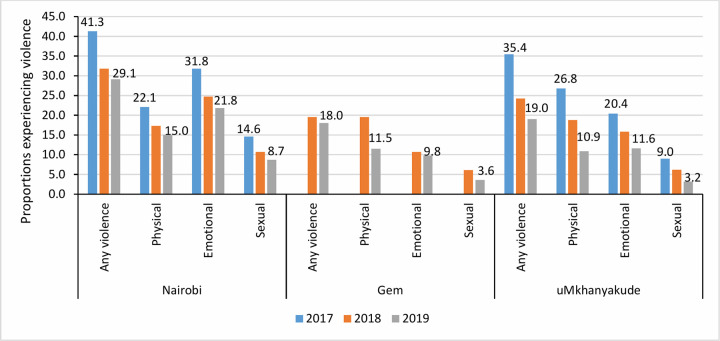
Violence trends among AGYW in Nairobi, Gem and uMkhanyakude between 2017 and 2019.

Fig 2 shows the levels of different forms of violence reported by AGYW over the evaluation period by age group. The proportion of AGYW who self-reported experiencing any form of violence was higher in Nairobi than in Gem and uMkhanyakude (e.g., 29% vs. 18% vs. 19%, respectively, in 2019) (**Fig 2**). In Nairobi and uMkhanyakude, a decline was observed in the proportions of AGYW reporting violence between 2017 and 2019 **(Fig 2).** The proportion who self-reported experiencing any form of violence fell from 41% to 29% in Nairobi, and from 35% to 19% in uMkhanyakude. Levels of violence reported remained stable in Gem at 18% in 2018 and 2019. Emotional and physical violence were the most commonly reported form of violence (in 2019, 22% and 15% in Nairobi; 12% and 11% in uMkhanyakude; 10% and 11% in Gem, respectively), while levels of sexual violence were under 10% in 2019. However, the proportion of AGYW self-reporting sexual violence was higher in Nairobi in 2017 (15%) and 2019 (9%) than in Gem and uMkhanyakude (3–6%). In Nairobi, a slightly higher proportion of AGYW aged 18–22 years reported experiences of violence compared to those aged 15–17 years, particularly for sexual violence (11% vs 7%). In Gem and uMkhanyakude, levels of violence reported by AGYW aged 13–17 and 18–22 years were similar.

### Impact of DREAMS on experience of violence

#### Descriptive results and multivariable logistic regression

**Table 2** presents the proportions reporting violence in 2019 by DREAMS invitation status, and adjusted odds ratios (aORs) for the associations between invitation to DREAMS and experience of violence. Proportions reporting recent violence were very similar among those invited to DREAMS and those not invited, with small absolute differences of 1–2 percentage points and the largest increase observed was 6% (e.g., 17% of those invited to DREAMS and 13% of those not invited reported physical violence in Nairobi).

**Table 2:** Association between DREAMS and experiences of violence among AGYW followed up in 2019 by site using conventional logistic regression.

Violence form	Setting	Never invited		Invited to DREAMS				
		N	n (%) with outcome	N	n (%) with outcome	Unadjusted model OR (95% CI)	Age-adjusted model OR (95% CI)	Fully Adjusted model OR (95% CI)
Any violence	Nairobi	224	69 (30.8)	628	179 (28.5)	0.9 (0.6–1.3)	0.9 (0.7–1.3)	0.9 (0.6–1.3)
	Gem	436	81(18.6)	582	102(17.5)	0.9 (0.7,1.3)	0.9 (0.7–1.3)	0.9 (0.6–1.2)
	uMkhanyakude	809	155 (19.2)	903	170 (18.8)	1.0 (0.8–1.3)	1.0 (0.8–1.3)	1.0 (0.8–1.3)
Emotional violence	Nairobi	224	52 (23.2)	628	134 (21.3)	0.9 (0.6–1.3)	0.9 (0.6–1.3)	0.9 (0.6–1.3)
	Gem	436	43(9.9)	582	57(9.8)	1.0 (0.7–1.5)	1.0 (0.7–1.5)	0.9 (0.6–1.4)
	uMkhanyakude	809	92 (11.4)	903	106 (11.7)	1.0 (0.8–1.4)	1.1 (0.8–1.4)	1.0 (0.7–1.4)
Physical violence	Nairobi	224	28 (12.5)	628	100 (15.9)	1.3 (0.9–2.1)	1.4 (0.9–2.2)	1.4 (0.9–2.3)
	Gem	436	50(11.5)	582	63(10.8)	0.9 (0.6–1.4)	0.9 (0.6–1.4)	0.8 (0.6–1.3)
	uMkhanyakude	809	90 (11.1)	903	97 (10.7)	1.0 (0.7–1.3)	1.0 (0.7–1.3)	0.9 (0.7–1.3)
Sexual violence	Nairobi	224	18 (8.0)	628	56 (8.9)	1.2 (0.6–2.5)	1.2 (0.7–2.1)	1.2 (0.7–2.1)
	Gem	436	20(4.6)	582	17(2.9)	0.6 (0.3–1.2)	0.6 (0.3–1.2)	0.7 (0.3–1.3)
	uMkhanyakude	809	28 (3.5)	903	35 (3.9)	1.1 (0.7–1.9)	1.1 (0.7–1.8)	1.2 (0.7–2.0)

Notes: Row percentages are presented; Fully adjusted models adjusted for age, site, ethnicity, level of education, schooling status, food insecurity, marital status, orphanhood, pregnancy history, sexual history at baseline; In Nairobi, age-adjusted model also adjusted for site.

Across all settings, there was very little evidence of an association between DREAMS and overall levels of violence: adjusted odds-ratio (aOR) of 0.9 (CI 0.6–1.3) in Nairobi; aOR 0.9 (CI 0.6–1.2) in Gem; and aOR 1.0 (CI 0.8–1.3) in uMkhanyakude. In Nairobi, there was weak evidence of greater odds of experiencing physical violence among DREAMS beneficiaries compared to non-beneficiaries (aOR 1.4, (CI 0.9–2.3)). **S5 Table** presents an expanded results of the logistic regression by invitation to DREAMS and age-group for the three study sites. For older AGYW in Gem, there was weak evidence of lower odds of experiencing any form of violence among DREAMS beneficiaries compared to non-beneficiaries (aOR 0.6, (CI 0.4–1.1), p = 0.09). Site was largely unattributed to any notable difference in findings (see online **S5 Table**).

In terms of other factors associated with experiences of any form of violence in the adjusted multivariable logistic regression models, violence among AGYW in Nairobi was associated with food insecurity (aOR 1.4, CI (1.0–2.0), p = 0.03), ever having sex (aOR 1.9, CI (1.1–3.3), p = 0.02), and ethnicity (more likely among the Luo and Luhya ethnic communities compared to other groups, p = 0.004) (**S6 Table**). In Gem, AGYW with lower socio-economic status were less likely to experience any violence than those with higher status (aOR 0.6, CI (0.3–1.1), p = 0.05). In uMkhanyakude, none of the characteristics examined were associated with violence.

### Estimated causal impact of DREAMS

**Fig 3:**
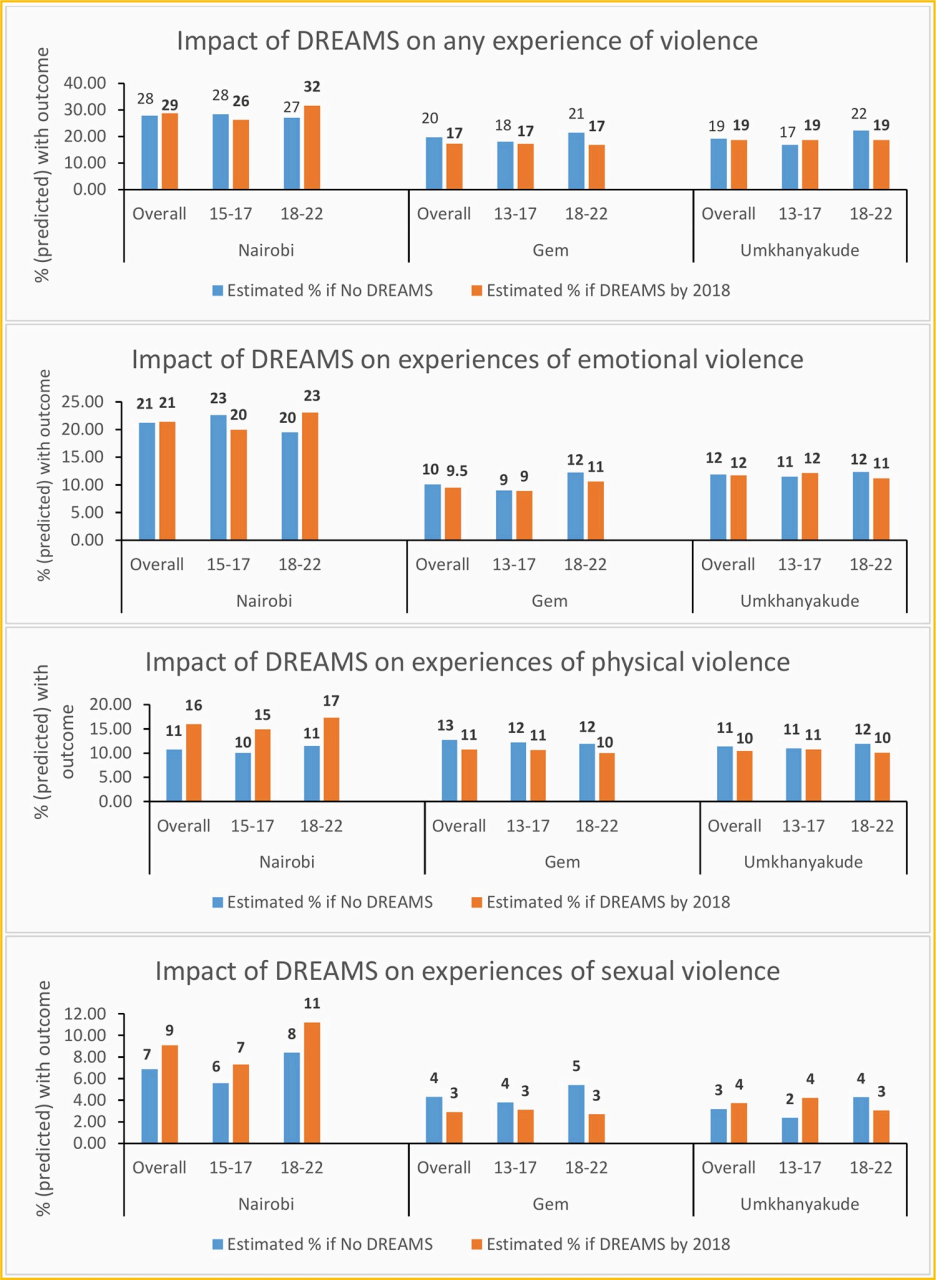
Estimated causal impact of DREAMS on different experiences of violence among AGYW in Nairobi, Gem, and uMkhanyakude.

The estimated causal effect of DREAMS on experiences of violence among AGYW is presented in **Table 3** and **Fig 3**. There was no evidence of a difference attributable to DREAMS in the overall levels of violence experienced in 2019. In Nairobi, it was estimated that proportions of AGYW who experienced any form of violence would be similar under the counterfactual scenarios that all AGYW were DREAMS beneficiaries and that none were DREAMS beneficiaries (28.7% compared to 27.8% respectively, difference +0.9% with CI (-6.5%, +7.7%)). For Gem, the equivalent percentages were 17.3% versus 19.7% (-2.4% (-7.6%, +2.8%)) and for uMkhanyakude 18.7% and 19.1% (-0.4% (-4.3%, +3.5%)). In Gem, among the older age group, there was a reduction of 4.7% estimated if all AGYW were DREAMS beneficiaries (16.8%) versus if none were DREAMS beneficiaries (21.4%), with weak statistical evidence to support this difference (CI (-12.8%, +3.3%)).

**Table 3:** Estimated causal effect of DREAMS on experiences of violence among AGYW followed up in 2019 by site.

Violence form	Setting	Age category	% with outcome in total study population	Estimated % with outcome if none benefit from DREAMS % (95% CI)	Estimated % with outcome if all benefit from DREAMS % (95% CI)	Risk difference % (95% CI)
Any violence	Nairobi	Overall	29.1	27.82 (22.44–34.60)	28.67 (25.17–32.46)	0.85 (-6.5–7.66)
		15–17	27.4	28.41 (20.30–38.24)	26.22 (21.94–30.97)	-2.19 (-13.4–7.53)
		18–22	31.2	27.11 (20.22–34.65)	31.60 (25.61–37.15)	4.49 (-4.83–13.47)
	Gem	Overall	18.5	19.7 (16.10–23.80)	17.3 (14.30–20.50)	-2.40 (-7.60–2.80)
		13–17	18.6	18.00 (13.10–22.70)	17.2 (13.10–21.30)	-0.80 (7.10–5.10)
		18–22	18.4	21.4 (15.20–28.30)	16.8 (11.90–21.80)	-4.70 (-12.80–3.30)
	uMkhanyakude	Overall	19.0	19.12 (16.18–22.11)	18.69 (15.98–21.47)	-0.42 (-4.32–3.54)
		13–17	18.1	16.78 (12.94–20.91)	18.70 (15.66–21.98)	1.92 (-3.11–7.04)
		18–22	20.1	22.26 (18.13–26.36)	18.68 (14.22–23.55)	-3.58 (-9.81–2.62)
Emotional violence	Nairobi	Overall	21.8	21.21 (15.90–26.84)	21.38 (18.18–24.46)	0.17 (-6.86–6.22)
		15–17	21.1	22.64 (14.08–30.45)	19.96 (15.91–23.98)	-2.68 (-11.54–6.28)
		18–22	22.7	19.50 (13.70–26.98)	23.08 (18.05–28.37)	3.58 (-5.66–11.28)
	Gem	Overall	10.9	10.10 (7.30–13.40)	9.50 (7.20–11.70)	0.60(4.40–3.20)
		13–17	10.7	9.00 (5.60–12.60)	8.90 (6.00–11.70)	-0.10 (-4.80–4.30)
		18–22	11.1	12.20 (7.30–17.50)	10.60 (7.00–14.80)	-1.70 (-8.80–4.70)
	uMkhanyakude	Overall	11.6	11.83 (9.38–14.5)	11.72 (9.48–14.01)	-0.11 (-3.45–3.26)
		13–17	11.8	11.49 (8.29–15.29)	12.12 (8.41–14.85)	0.63 (-3.90–5.13)
		18–22	11.2	12.29 (9.26–15.46)	11.18 (7.56–15.17)	-1.11 (-6.22–4.12)
Physical violence	Nairobi	Overall	15.0	10.69 (7.29–15.35)	15.98 (12.90–19.05)	5.29 (0.20–10.00)
		15–17	14.2	10.04 (4.88–15.96)	14.88 (11.36–18.59)	4.84 (-2.05–11.17)
		18–22	16.0	11.47 (6.44–18.50)	17.31 (13.04–21.75)	5.83 (-1.67–12.89)
	Gem	Overall	10.9	12.70 (9.60–16.20)	10.70 (8.10–13.50)	-1.90 (-6.50–2.40)
		13–17	11.2	12.20 (8.20–16.30)	10.60 (7.40–14.10)	-1.60 (-7.00–3.40)
		18–22	10.6	11.90 (6.80–17.30)	10.00 (6.30–14.40)	-1.90 (-8.30–4.90)
	uMkhanyakude	Overall	10.9	11.35 (8.95–13.66)	10.46 (8.37–12.67)	-0.89 (-3.97–2.26)
		13–17	11.0	10.97 (7.61–14.35)	10.73 (8.19–13.43)	-0.24 (-4.48–3.96)
		18–22	10.8	11.87 (8.70–15.02)	10.09 (6.68–13.72)	-1.78 (-6.44–3.13)
Sexual violence	Nairobi	Overall	8.7	6.85 (4.06–10.56)	9.08 (6.79–11.48)	2.23 (-2.13–5.99)
		15–17	7.1	5.56 (2.03–11.02)	7.31 (4.74–9.96)	1.74 (-3.87–5.95)
		18–22	10.6	8.38 (4.08–13.43)	11.20 (7.64–15.45)	2.82 (-3.37–8.78)
	Gem	Overall	3.9	4.30 (2.50–6.40)	2.90 (1.50–4.30)	-1.40 (-3.90–0.90)
		13–17	3.8	3.80 (1.80–6.40)	3.10 (1.40–5.10)	-0.70 (-3.60–2.20)
		18–22	4.0	5.40 (2.20–9.30)	2.70 (0.90–5.10)	-2.70 (-6.90–1.10)
	uMkhanyakude	Overall	3.7	3.18 (1.96–4.56)	3.72 (2.62–5.09)	0.54 (-1.21–2.43)
		13–17	3.8	2.37 (0.83–4.05)	4.22 (2.72–5.87)	1.85 (-0.37–4.14)
		18–22	3.5	4.28 (2.33–6.18)	3.05 (1.33–5.33)	-1.22 (-3.97–1.76)

There were some notable findings by violence form. In Nairobi, it was estimated that proportions of AGYW who experienced recent physical violence would increase from 10.7% to 16.0% if all AGYW were DREAMS beneficiaries (difference +5.3% with CI (0.2%,10.0%). This rise was evident in both the younger and older age groups, with weak statistical evidence in sub-analyses by age-group. In Gem, there was borderline evidence of a decrease in experiences of sexual violence, attributable to DREAMS, among older AGYW (difference -2.7% with CI (-6.9%, +1.1%). For all other forms of violence and age sub-groups, there was no evidence of a causal impact of DREAMS.

Compared to the primary analyses presented above, results were very similar in sensitivity analyses that used alternative approaches to control for confounding (**S18 Table**).

## Discussion

This study assessed the impact of DREAMS’ multi-component intervention on experiences of violence among AGYW in ‘real-world’ non-trial conditions in rural and urban settings in Kenya and South Africa. Our results revealed that recent violence perpetrated by males was common among AGYW. Applying a causal inference analysis, we found little evidence of DREAMS’ impact on such experiences within three years of implementation.

Across the three diverse study settings in 2019, about 20–30% of AGYW reported experiencing at least one form of violence inflicted by a man in the previous 12 months. These estimates are broadly consistent with the WHO Africa regional estimates for 2018 of 36% prevalence of lifetime IPV and/or non-partner violence among all women aged 15–49, and country-specific prevalence estimates of recent IPV among ever- partnered women aged 15–49 years for Kenya (23%) and South Africa (13%) [3]. Recent studies conducted in Kenya, South Africa, and other sub-Saharan African countries indicate persistently high levels of GBV among women [4–7]. Levels of violence reported in our study were highest in informal settlements in Nairobi. An earlier cross-sectional study, conducted in the same setting, to examine the association between violence victimization and expectations for achieving aspirations, reported that one-third of adolescent girls aged 11–15 had experienced at least one form of violence [6]. A similarly high level of recent IPV (30%) was reported at baseline among young women aged 18–30 living in urban settlements in Durban, South Africa, before intervention implementation [60]. High vulnerability driven by livelihood insecurity and poor access to services in urban informal settlements has been linked to poor health outcomes, including exposure to violence and HIV, which may partly explain the high prevalence of violence we observed in Nairobi [6,60,61]. This interpretation is further supported by higher levels of violence reported among those who were food insecure or of lower socio-economic position in our Kenyan analyses.

We found little evidence of impact of the multi-component DREAMS intervention package on experiences of violence across our study settings. Challenges with intervention implementation may be one of the reasons explaining the limited impact. For instance, a study conducted in South Africa to assess the effect of community mobilisation on men’s IPV perpetration reported contextual challenges as a barrier to successful implementation [62]. This is consistent with process evaluation reports which have reported contextual factors such as loss to follow-up of AGYW who either migrated or were unable to keep up with the interventions; or the volunteers participating in mentoring sessions due to better economic opportunities. Furthermore, there were some interruptions in programming in the first year of implementation in Kenya due to political interruptions brought about by national elections. Among the DREAMS core package of interventions, those targeting GBV reduction include parenting/ caregiver programs, school-based HIV and violence prevention, and community education and norms change [35]. Available evidence indicates low uptake (<10%) of parenting/caregiver and community mobilisation interventions targeted at young men and community members, in contrast to the very high levels of participation of AGYW in girl-centered interventions [51]. Interventions targeted at the community and partners of AGYW have been shown to be effective at reducing GBV by changing attitudes and promoting equitable social norms against GBV, especially among men. For example, the ‘Raising Voices SASA!’ kit and Safe Homes and Respect of Everyone (SHARE) interventions implemented in Uganda were associated with lower IPV incidence and more supportive community responses to female victims [34,63]. However, these examples come from trial conditions with high fidelity, and more time and training may be needed to embed and adapt community-based norms-change and GBV interventions that were newly introduced into these settings with DREAMS investments [34,52,54,64].

The limited effect of DREAMS’ influence on gender attitudes and norms among men is evident in related analyses, which found little change over time in attitudes among large, representative samples of men in Gem and Nairobi [65]. Meanwhile, the proportion of AGYW with gender-equitable attitudes (toward sexual relationships and gender-based violence) increased annually during the DREAMS implementation period. Evidence from a general population-based survey done in the same community in Nairobi during 2019 also showed that around a fifth of young men in partnerships admitted to perpetration of violence, and that this was associated with less equitable attitudes towards gender norms [66]. Shifting ingrained inequitable social norms is recognised as challenging, for example due to resistance from patriarchal and traditional structures, and may take longer than the 2–3 year period of our evaluation. Recommendations for practitioners designing social norms interventions have recently been outlined, which may aid the design and adaptation of programmes in future [67]. The drawbacks of interventions that treat norms in isolation have been emphasized, and the importance of integrating norms change approaches, and those aimed at tackling GBV, with other individual, social, and structural approaches, as complex interventions such as DREAMS have aimed to do [67].

While some gender-transformative interventions aimed at youth in other contexts have been shown to be effective at addressing inequitable social norms and reducing GBV, other studies have shown limited impact [30,60,68]. For example, the Stepping Stones and Creating Futures intervention in South Africa combining educational gender-transformative curricula and life-skills training to strengthen livelihoods did not impact on experiences of IPV among young women after two years of implementation, although the intervention was effective at reducing men’s self-reported perpetration of IPV [29]. Although the Stepping Stones and Creating Futures intervention included participatory sessions with young out-of-school men, it did not include a broad community-based element as other more successful interventions such as SASA! have done [34]. Yet, there are also examples of interventions with a narrower focus, such as PREPARE in South Africa which incorporated school-based HIV and violence prevention education for girls and boys, health services, and safety programme components, that have been successful in reducing IPV victimization among adolescent girls [69]. While the evidence is, therefore, unclear on the necessity of including community-norms change components to reduce GBV among AGYW, this will be important in reaching the Sustainable Development Goals (SDGs) for health and gender equality. Indeed, a recent systematic review of evidence-based gender-transformative health interventions for children and young people globally concluded that tackling the societal and structural elements of restrictive gender norms, to elicit systemic change through social participation and multi-level, multi-sectoral action, was key to achieving the SDGs [70].

We observed an increase in reported physical violence due to DREAMS in Nairobi, and a trend in this direction for other violence forms in the same setting. One possible explanation is an increase in awareness, acknowledgement and disclosure of personal experiences of violence in this setting, and therefore increased reporting of violence, as a result of exposure to violence prevention education and empowerment curricula from the DREAMS intervention package. Another Nairobi-based study reported an increase in disclosure of experiences of sexual assault to others following an empowerment and self-defence skills intervention [71]. However, this would not explain the absence of an increase in other forms of violence in Nairobi (sexual and emotional), or increased reporting of violence among DREAMS beneficiaries in the other two settings. Alternatively, the finding may reflect a true increase in the violence experienced by DREAMS beneficiaries in Nairobi, as a result of participating in DREAMS. This may be explained using the backlash theory where there is resistance to violence-prevention interventions against women and girls, which may include increased risk of more violence [72]. For instance, in a related process evaluation of DREAMS in Nairobi, mentors reported conflict with some male partners who resisted the empowerment of their female partner in DREAMS safe spaces [52]. There are other examples of interventions seeking to reduce violence against young women in which impacts have been mixed across different violence outcomes, and an unintended increase in violence has been reported [73]. This phenomenon has been described in the social sciences literature, with the rationale that a man may inflict violence to re-impose authority over his wife/partner [73–75]. It is an unintended consequence of empowerment programmes which must be carefully considered in programme planning, to minimise harm and maximise safety of participants. It is more likely to occur in programmes that do not effectively reach the perpetrators of violence. Finally, in-depth analysis of qualitative data should be conducted to understand this exceptional finding, and whether it may be spurious.

Our results were generally consistent across age-groups of AGYW, except for Gem where there was a pattern of reduced violence among older but not younger AGYW. This is consistent with our related analysis of equitable attitudes towards violence-related gender norms, which showed more equitable norms in the older compared to the younger age group [65].

Descriptive analyses of trends over time suggested a large decline in the levels of violence between 2017 and 2019 in Nairobi and uMkhanyakude. The findings can be supported by the aging out theory that indicates that most GBV exposure or perpetration tends to reduce over time due to various reasons. A possible explanation for this observation in Nairobi is the differential cohort attrition by violence at enrolment, such that those who had reported violence at enrolment were more likely to be LTFU by 2019 than those who had not experienced violence at enrolment, which may have artificially deflated the proportions experiencing violence by 2019. In uMkhanyakude, the decline does not appear to be explained by cohort attrition. Here, it is possible that other (non-DREAMS) outreach campaigns against GBV in the community, organised by the municipality, could have influenced ‘background’ levels of violence experienced and reported by young women in the community. Additionally, the DREAMS violence-prevention interventions implemented in uMkhanyakude, Vhutshilo and Stepping Stones [36,38], targeted the wider communities and DREAMS may have heralded a wider awareness of GBV and social norms change.

Key strengths of this study were the representative selection of AGYW from three established DSS sites, harmonised data collection, and high cohort retention across the study settings. While cohort retention was high, it was differential by AGYW characteristics, possibly leading to a small degree of selection bias. A further limitation is that violence outcomes were self-reported and AGYW may have under-reported their experiences, particularly of sexual violence, due to feelings of shame or fear of blame or repercussion. However, overall experiences of recent violence were commonly reported and in-line with other reports in the literature. There is also a chance that the trained field interviewers may have been perceived to be DREAMS program staff rather than independent evaluation staff by respondents, potentially causing some social desirability bias in their responses. A degree of bias may also have been introduced if DREAMS invitees were more, or less, likely to report experiences of violence, as a result of their experience in the programme. Misclassification of exposure status could also have occurred if some AGYW did not report themselves as DREAMS invitees, potentially underestimating the proportion of DREAMS beneficiaries. This is unlikely in Kenyan settings where a single DREAMS IP invited AGYW to participate in DREAMS, although it could plausibly have occurred in uMkhanyakude. Furthermore, the tool did not collect information on other avenues with which violence against AGYW may have been perpetrated such as through technological abuse, stalking, financial abuse etc. which may also be a potential source of misclassification of exposure. Our results may not be generalizable to all DREAMS districts, but the diverse implementation contexts can offer insights for other settings implementing DREAMS.

## Conclusions

Our study contributes important evidence to understanding whether complex interventions have an impact on reducing experiences of violence among AGYW in real-world non-trial contexts. We have shown that recent experiences of GBV were common among AGYW, especially in urban Kenya, and DREAMS had limited impact on reducing violence after 2–3 years of implementation. While there are a handful of efficacious multi-component interventions that reduce GBV towards AGYW from trial settings, there is need for more investment in implementation research to bridge the findings from efficacy to population-level effectiveness. Furthermore, tailoring programs to work in close partnership with communities could help address the differing perspectives and needs of men and women, and different age groups to improve program outcomes.

## Supporting information

S1 Table Proportions of AGYW retained in the study vs lost to follow up by 2019, by AGYW characteristics in Nairobi. (XLSX)

S2 Table Proportions of AGYW retained in the study vs lost to follow up by 2019, by AGYW characteristics in Gem. (XLSX)

S3 Table Proportions of AGYW retained in the study vs lost to follow up by 2019, by AGYW characteristics in uMkhanyakude. (XLSX)

S4 Table Characteristics of DREAMS beneficiaries and non-beneficiaries at time of cohort enrolment by invitation to DREAMS in Nairobi, Gem and uMkhanyakude. (XLSX)

S5 Table Association between DREAMS and experiences of violence among AGYW followed up in 2019 by site and age group using conventional logistic regression. (XLSX)

S6 Table Association between invitation to DREAMS and any experience of violence among AGYW in Nairobi using conventional logistic regression. (XLSX)

S7 Table Association between invitation to DREAMS and any experience of violence among AGYW in Gem using conventional logistic regression. (XLSX)

S8 Table Association between invitation to DREAMS and any experience of violence among AGYW in uMkhanyakude using conventional logistic regression. (XLSX)

S9 Table Association between invitation to DREAMS and physical violence among AGYW in Nairobi using conventional logistic regression, 13–22 year-olds. (XLSX)

S10 Table Association between invitation to DREAMS and physical violence among AGYW in Gem using conventional logistic regression, 13–22 year-olds. (XLSX)

S11 Table Association between invitation to DREAMS and physical violence among AGYW in uMkhanyakude using conventional logistic regression, 13–22 year-olds. (XLSX)

S12 Table Association between invitation to DREAMS and emotional violence among AGYW in Nairobi using conventional logistic regression, 13–22 year-olds. (XLSX)

S13 Table Association between invitation to DREAMS and emotional violence among AGYW in Gem using conventional logistic regression, 13–22 year-olds. (XLSX)

S14 Table Association between invitation to DREAMS and emotional violence among AGYW in uMkhanyakude using conventional logistic regression, 13–22 year-olds. (XLSX)

S15 Table Association between invitation to DREAMS and sexual violence among AGYW in Nairobi using conventional logistic regression, 13–22 year-olds. (XLSX)

S16 Table Association between invitation to DREAMS and sexual violence among AGYW in Gem using conventional logistic regression, 13–22 year-olds. (XLSX)

S17 Table Association between invitation to DREAMS and sexual violence among AGYW in uMkhanyakude using conventional logistic regression, 13–22 year-olds. (XLSX)

S18 Table Sensitivity analyses on the effect of DREAMS on experiences of violence among AGYW. (XLSX)
